# Hyperglycaemia up-regulates placental growth factor (PlGF) expression and secretion in endothelial cells via suppression of PI3 kinase-Akt signalling and activation of FOXO1

**DOI:** 10.1038/s41598-021-95511-8

**Published:** 2021-08-11

**Authors:** Samir Sissaoui, Stuart Egginton, Ling Ting, Asif Ahmed, Peter W. Hewett

**Affiliations:** 1 Institute of Cardiovascular Sciences, College of Medical and Dental Sciences, University of Birmingham, Edgbaston, Birmingham, B15 2TT UK; 2 Multidisciplinary Cardiovascular Research Centre, University of Leeds, Leeds, LS2 9JT UK; 3 MyrZyme Therapeutics Ltd, Faraday Wharf, Innovation Birmingham Campus, Holt Street, Birmingham, B4 4BB UK; 4 School of Health Sciences, University of Southampton, Southampton, SO17 1BJ UK; 5 Present Address: Arima Genomics, 6404 Nancy Ridge Drive, San Diego, CA 92121 USA

**Keywords:** Cell biology, Cell signalling, Growth factor signalling, Cardiovascular diseases

## Abstract

Placenta growth factor (PlGF) is a pro-inflammatory angiogenic mediator that promotes many pathologies including diabetic complications and atherosclerosis. Widespread endothelial dysfunction precedes the onset of these conditions. As very little is known of the mechanism(s) controlling PlGF expression in pathology we investigated the role of hyperglycaemia in the regulation of PlGF production in endothelial cells. Hyperglycaemia stimulated PlGF secretion in cultured primary endothelial cells, which was suppressed by IGF-1-mediated PI3K/Akt activation. Inhibition of PI3K activity resulted in significant PlGF mRNA up-regulation and protein secretion. Similarly, loss or inhibition of Akt activity significantly increased basal PlGF expression and prevented any further PlGF secretion in hyperglycaemia. Conversely, constitutive Akt activation blocked PlGF secretion irrespective of upstream PI3K activity demonstrating that Akt is a central regulator of PlGF expression. Knock-down of the Forkhead box O-1 (FOXO1) transcription factor, which is negatively regulated by Akt, suppressed both basal and hyperglycaemia-induced PlGF secretion, whilst FOXO1 gain-of-function up-regulated PlGF in vitro and in vivo. FOXO1 association to a FOXO binding sequence identified in the PlGF promoter also increased in hyperglycaemia. This study identifies the PI3K/Akt/FOXO1 signalling axis as a key regulator of PlGF expression and unifying pathway by which PlGF may contribute to common disorders characterised by endothelial dysfunction, providing a target for therapy.

## Introduction

Diabetes mellitus is associated with both central and peripheral vasculopathies, notably diabetic retinopathy^1^. Patients also have an increased incidence of cardiovascular disease (CVD), susceptibility to associated complications such as plaque rupture and atherothrombosis, and approximately 75% die from vascular-related disease^1,2^. Intensive management of blood glucose significantly reduces the risk of microvascular complications^3^. Excessive superoxide anion generation in the mitochondria was proposed as the common mechanism underlying vascular injury in response to hyperglycaemia, although other factors such as insulin resistance, diabetic dyslipidemia, and advanced glycation end products contribute in type-2 diabetes^2,4,5^. Alterations in endothelial function precede the onset of diabetic complications, and are characterised by reduced endothelial nitric oxide (NO) synthase activity, elevated levels of inflammatory and coagulation markers, and increased endothelial cell apoptosis^6^.

The phosphoinositide-3’-kinase (PI3K)/Akt (PKB) pathway is important for both mature endothelial cell and progenitor cell function^7,8^ and its activity is reduced in both type-1 and type-2 diabetes mellitus^6^. Several mechanisms may contribute to the down-regulation of PI3K signalling in type-2 diabetes including the up-regulation of TBR3, a CDC25 binding protein homolog, which directly inhibits Akt activity^9^, and induction of the PI3K antagonist PTEN (phosphatase and tensin and homologue deleted on chromosome 10)^10^. Alternatively, there may be reduced levels of ligands and/or receptor activity coupled to this pathway.

Placenta growth factor (PlGF) is a member of the vascular endothelial growth factor (VEGF) family that mediate vascular development and homeostasis. Like VEGF, PlGF is a pleiotropic cytokine that promotes angiogenesis, vascular remodelling, cell survival and inflammation^11^. PlGF acts via the VEGF receptor-1 (VEGFR-1) and neuropilin accessory receptors, but leads to the phosphorylation of different VEGFR-1 tyrosine residues and gene expression to those activated by VEGF-A^12^. The expression of PlGF is low, or absent, in most healthy adult tissues and its activity is reported to be confined to pathological situations such as diabetes, atherosclerosis, arthritis and cancer making it an attractive target for therapy^11^.

Our laboratory reported high vitreous PlGF levels in patients with proliferative diabetic retinopathy^13^, which was confirmed by others^14,15^. The intravitreous or systemic delivery of PlGF in rodents disrupts retinal barrier function and increases vascular leakage in a VEGFR-1-dependent manner, mimicking diabetic retinopathy^15,16^. In addition, administration of soluble VEGFR-1 receptor (sFlt-1), which sequesters both PlGF and VEGF, prevents diabetic retinopathy in a spontaneous rat model^11,17^. Furthermore, delivery of an anti-PlGF antibody in a mouse diabetic retinopathy model reduced vascular leakage, inflammation and fibrosis^18^. Elevated circulating PlGF levels are associated with vascular inflammation and adverse outcome in patients with acute coronary syndrome^19^, severity of metabolic syndrome^20^, development of type-2 diabetes^21^ and childhood obesity^22^. Increased PlGF expression in atherosclerotic plaques correlate with plaque instability, inflammation and intimal angiogenesis^23^. In atherosclerosis-prone apolipoprotein E deficient (ApoE^−/−^) mice, PlGF is up-regulated in early lesions and loss of PlGF reduces plaque size and macrophage content^24,25^. Interestingly, PlGF null mice also have a lower fat content when maintained on a high-fat diet^26^. However, the mechanism(s) by which PlGF expression is up-regulated in these pathologies remain unknown. In this study we show that manipulation of glucose concentration, PI3K or Akt activity leads to consistent changes in endothelial cell PlGF expression and release, which are dependent on FOXO1 transcription factor activity. The identification of the involvement of the PI3K/Akt/FOXO1 pathway in the control of PlGF expression may have important implications for endothelial homeostasis and dysfunction, and therefore represent a target for therapy in vascular disorders.

## Results

### PlGF expression is modulated by hyperglycaemia in endothelial cells

**Figure 1:**
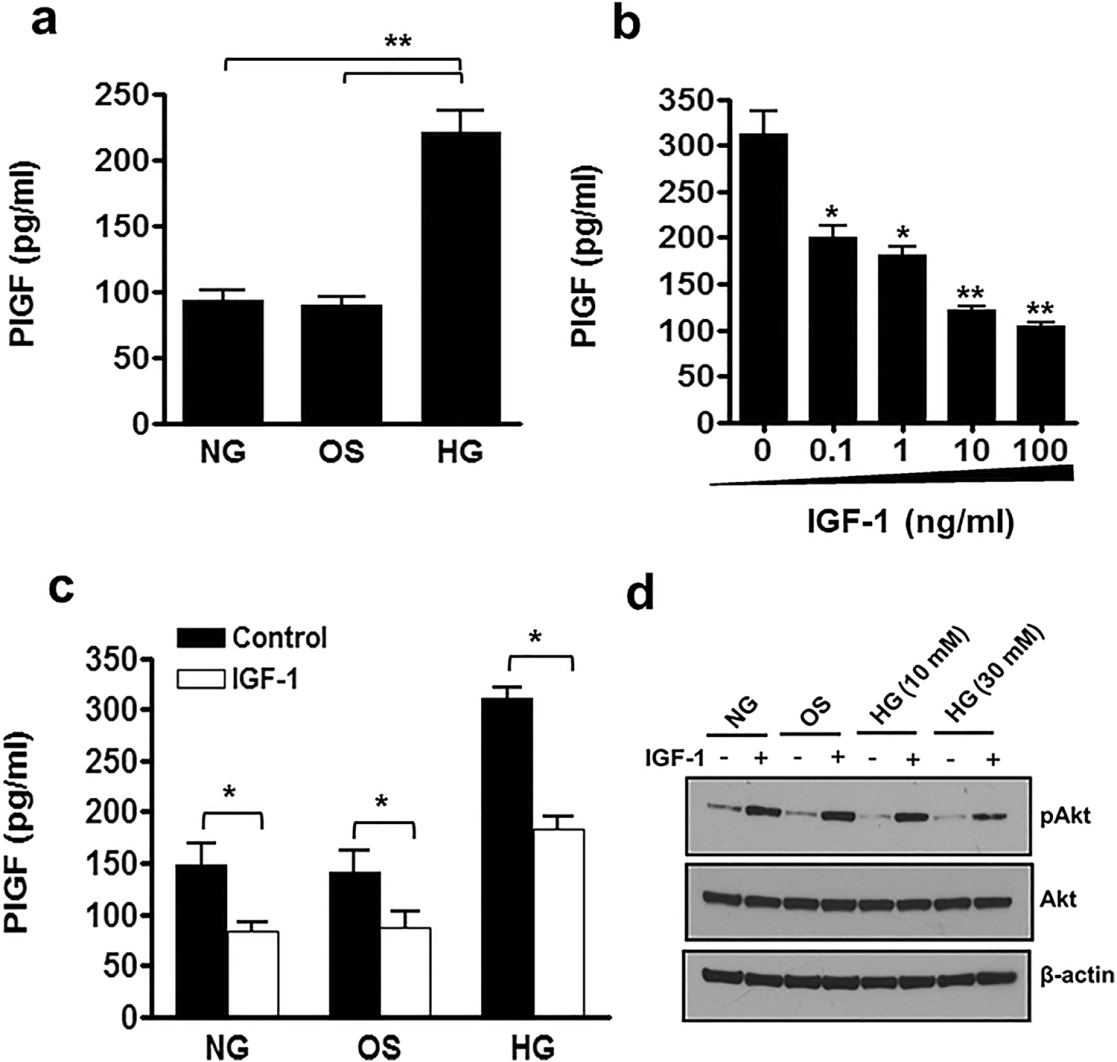
Hyperglycaemia suppresses Akt activity and promotes PlGF secretion in endothelial cells. (**a**) Confluent endothelial cells cultured for 24 h in either normolycaemia (NG = 5 mM d-glucose), hyperglycaemia (HG = 30 mM d-glucose) or osmolarity control (OS = 5 mM d-glucose + 25 mM l-glucose) medium, and the level of PlGF secretion measured by ELISA. (**b**) Confluent HUVEC stimulated with increasing concentrations of IGF-1 in medium containing 10% FCS for 24 h and PlGF secretion measured by ELISA. (**c**) HUVEC were cultured in medium containing either NG, HG or OS control in the presence of IGF-1 (10 ng/ml) for 24 h and PlGF secretion quantified by ELISA. (**d**) Confluent HUVEC were maintained in NG, HG or OS control conditions and then stimulated with IGF-1 (10 ng/ml) or vehicle for 10 min and cell lysates Western blotted for phospho-Akt^Ser473^ (pAkt^Ser473^), total Akt and β-actin. The unedited blot images are presented in Supplementary Figure S5. Results are the mean ± SEM of 3 or more independent experiments; Student’s t-test *P < 0.05, **P < 0.01.

Elevated circulating PlGF has recently been identified in insulin resistance, metabolic syndrome, vascular inflammation and is an independent marker of the future development of type-II diabetes^19–22^. To determine whether hyperglycaemia up-regulates the expression of PlGF, we incubated confluent primary human endothelial cells in medium containing 30 mM d-glucose for 24 h and measured the level of PlGF released into the medium by ELISA. Hyperglycaemia significantly up-regulated PlGF release from HUVEC (Fig. 1A) and HAEC (Fig. S1) compared with cells maintained under normal glucose levels (5 mM d-glucose) or osmolarity controls (25 mM l-glucose + 5 mM d-glucose). As hyperglycaemia is reported to suppress PI3K signalling in many cell types, including endothelial cells^26^ we examined the effect of the established PI3K activators, IGF-1 and insulin on PlGF expression. The basal level of PlGF produced by endothelial cells was reduced in a concentration-dependent manner by IGF-1 (Fig. 1B) and insulin (1 mM) led to a 31% decrease in PlGF secretion (data not shown), indicating that PlGF release is negatively regulated by PI3K pathway activity in endothelium. Furthermore, addition of IGF-1 (10 ng/ml) significantly reduced the level of PlGF release in endothelial cells grown under both hyperglycaemic and normoglycaemic conditions (Fig. 1C and Fig. S1). The inhibitory effect of hyperglycaemia on PI3K activity in HUVEC and its modulation with IGF-1 (10 ng/ml) were demonstrated by Western blotting for phoshorylation of Akt at Serine 473 (pAkt^Ser473^). The level of activity (pAkt^Ser473^) was found to be decreased by hyperglycaemia under both basal conditions and following IGF-1 stimulation in comparison with cells in normoglycaemia (Fig. 1D).

Up-regulation of PlGF expression was reported to be dependent on endogenous VEGF in bovine retinal endothelial cells^27^. To determine whether endogenous VEGF was involved in the observed up-regulation of PlGF in hyperglycaemia, VEGF was knocked-down in HUVEC prior to exposure to 30 mM d-glucose (Fig. S2). Surprisingly, knock-down of endogenous VEGF expression was found to promote PlGF mRNA expression and secretion under control conditions (Fig. S2A-C) and in the presence of hyperglycaemia indicating that PlGF production in hyperglycaemia is not secondary to the induction of endogenous VEGF (Fig. S2D). This also consistent with an observed decrease in Akt phosphorylation 24 h after the knock-down of endogenous VEGF (data not shown).

### PI3K signalling negatively regulates PlGF expression in endothelial cells

**Figure 2:**
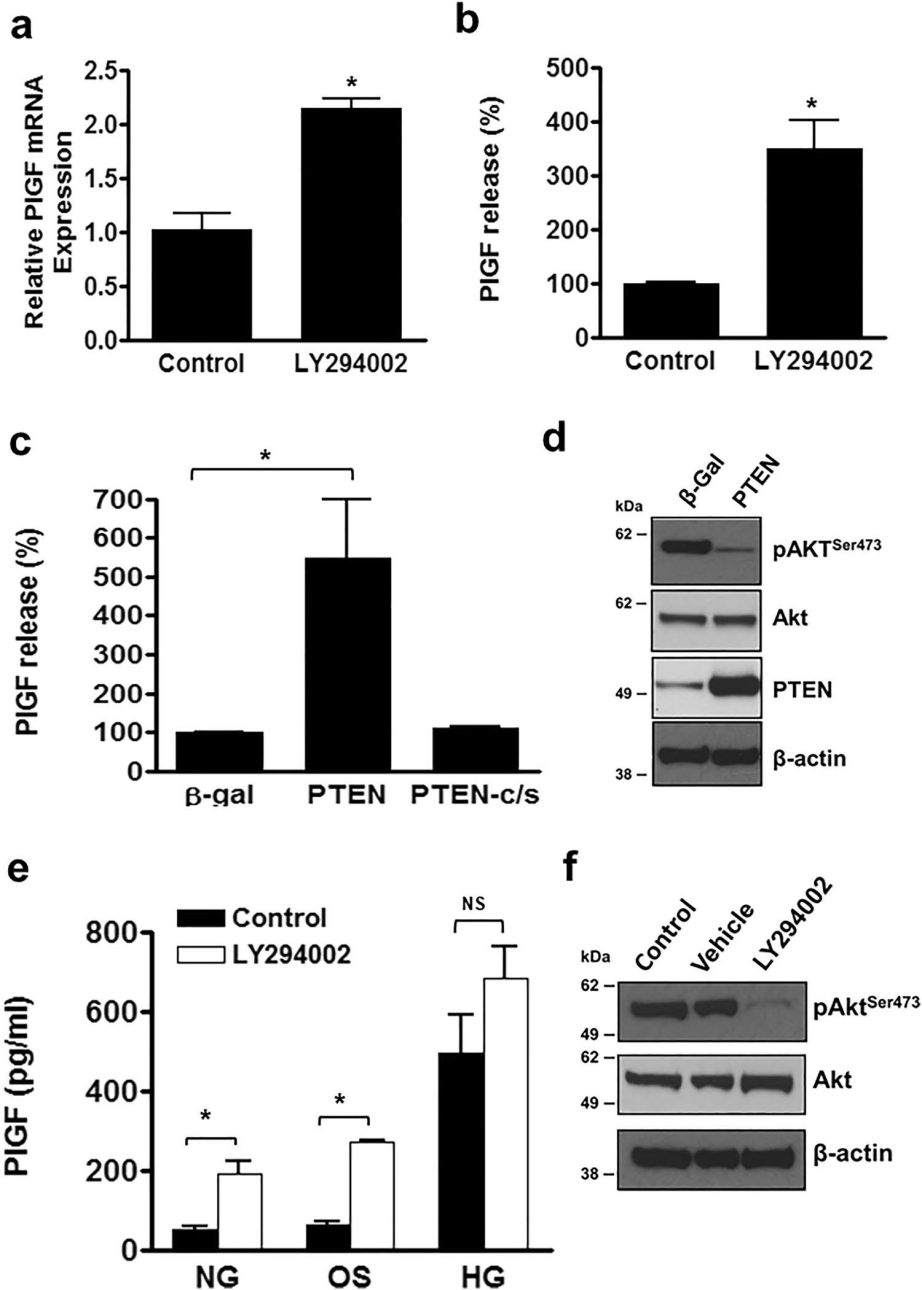
Inhibition of the PI3K pathway promotes PlGF expression and secretion. Confluent HUVEC pre-treated with LY294002 (20 µM) and incubated for (**a**) 8 h or (**b**) 24 h and PlGF mRNA quantified by qPCR and protein secretion by ELISA. (**c**) HUVEC were infected overnight with adenoviruses encoding PTEN, mutant PTEN (PTEN-c/s), or β-galactosidase (β-gal). Following 24-h incubation (**c**) PlGF levels in the culture medium were quantified by ELISA. (**d**) Western blots confirming inhibition of Akt activity in HUVEC following Ad-PTEN infection. (**e**) HUVEC treated with LY294002 or the vehicle and incubated for 24 h in normoglycaemia (NG = 5 mM d-glucose), hyperglycaemia (HG = 30 mM d-glucose), or osmolarity control (OS = 5 mM d-glucose + 25 mM l-glucose), and PlGF measured in supernatants by ELISA. (**f**) Representative Western blots of the HUVEC lysates following treatment of HUVEC with LY294002 or the vehicle for one hour showing pAkt^Ser473^, total Akt with β-actin as a loading control. The unedited blot images are presented in Supplementary Fig. S5. Results are the mean ± SEM of 3 or more experiments (n > 6), Student t-test; *P < 0.05, **P < 0.01.

To establish the involvement of the PI3K pathway in controlling PlGF expression, HUVEC were incubated with the PI3K inhibitor, LY294002, and PlGF expression and release examined by real-time qPCR and ELISA. Blockade of PI3K resulted in a significant increase in both PlGF mRNA and protein release (Fig. 2A,B). In addition, the use of recombinant adenoviruses encoding the PI3K antagonist, PTEN, produced a similar increase in PlGF release from HUVEC, whilst cells infected with a catalytically inactive PTEN mutant (PTENc/s) were not affected (Fig. 2C). Western blotting was performed to confirm that LY294002 treatment and PTEN over-expression led to a decrease in the activation of the downstream PI3K effector, Akt (Fig. 2D,F). This was demonstrated by the greatly reduced levels of phosphorylation of Akt serine 473 (pAkt^Ser473^). Furthermore, no further increase in PlGF release was observed from HUVEC cultured in medium containing 30 mM d-glucose following pre-treatment with LY294002, indicating that hyperglycaemia stimulates PlGF expression via its inhibitory action on the PI3K pathway (Fig. 2E).

### Akt is a negative regulator of PlGF expression in endothelial cells

**Figure 3:**
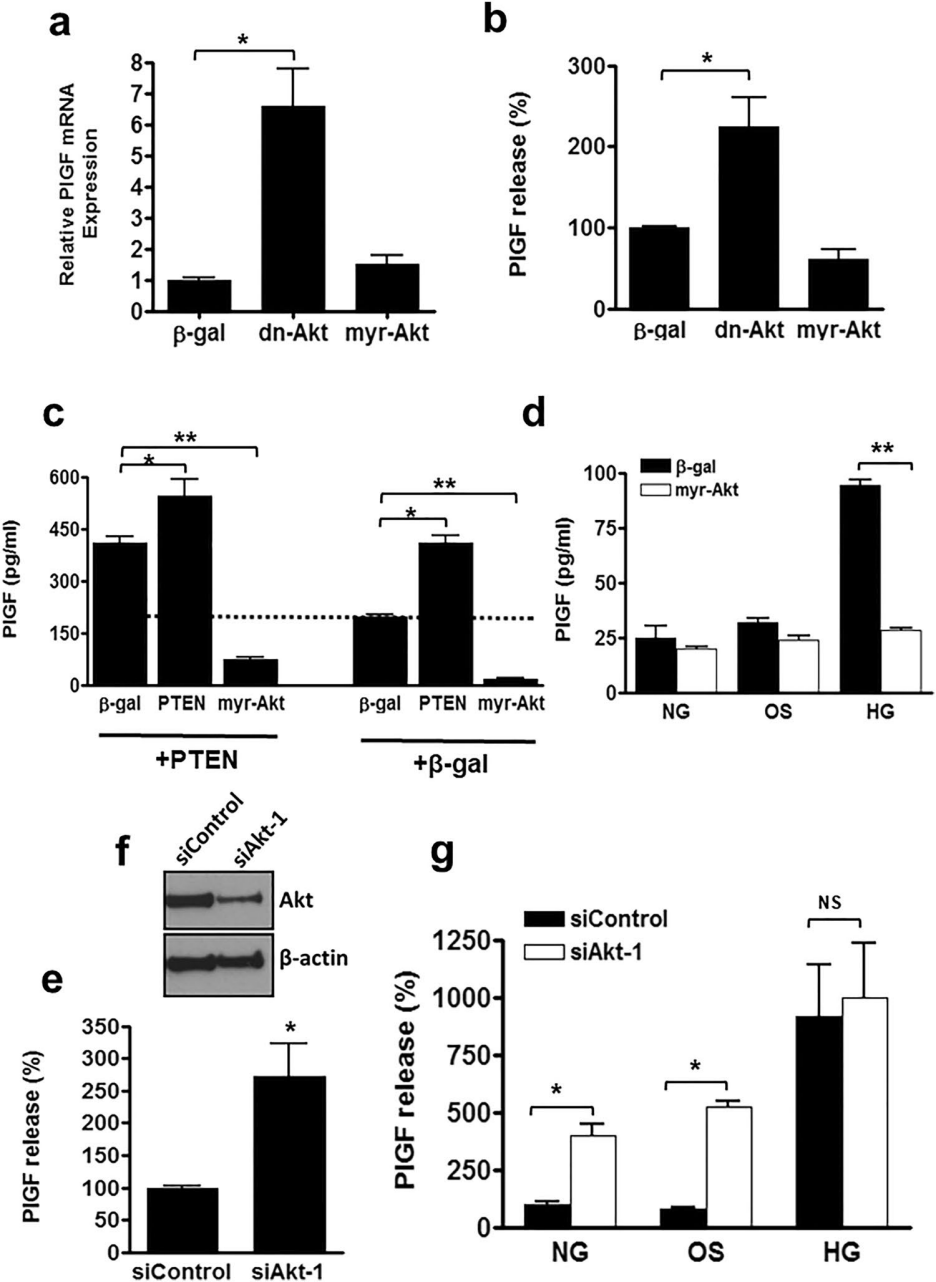
Inhibition of Akt activity increases PlGF production. HUVEC were transduced with adenoviruses encoding dominant-negative Akt (dn-Akt), constitutively active Akt (myr-Akt) and β-galactosidase (β-gal). Following (**a**) 8 h and (**b**) 24-h incubation PlGF expression and secretion was quantified by qPCR and ELISA. (**c**) HUVEC were co-infected with combinations of PTEN, myr-Akt and β-gal (control) adenoviruses overnight. After 24 h PlGF secretion was quantified by ELISA. (**d**) HUVEC were transduced with myr-Akt or β-gal and incubated in normoglycaemia (NG = 5 mM d-glucose), hyperglycaemia (HG = 30 mM d-glucose) or osmolarity control (OS = 5 mM d-glucose + 25 mM l-glucose), and PlGF release into the culture medium measured by ELISA. (**e - g**) HUVEC were electroporated with siRNA targeted to Akt-1 (siAkt) or control siRNA (siControl). (**e**) PlGF measured after 24 h in cell supernatants and (**f**) the cell lysates Western blotted for Akt and β-actin (unedited blot images in Supplementary Figure S5). (**g**) Cells were incubated in NG, HG or OS for 24 h and PlGF release measured by ELISA. Results are the mean ± SEM of 3 or more experiments (n = ≥ 6); Student’s t-test, *P < 0.05, **P < 0.01.

To determine whether PI3K acts via Akt to regulate PlGF expression, endothelial cells were infected with adenoviruses encoding either dominant-negative (dn-Akt), or constitutively active myristolated (myr-Akt) mutants of Akt. Following infection of HUVEC with dn-Akt adenoviruses (*see* Fig. S3), the levels of PlGF mRNA and protein released into the culture medium increased significantly compared with cells treated with control virus encoding β-galactosidase (β-gal) (Fig. 3A,B). To further establish the central role of Akt in the regulation of PlGF expression, HUVEC were infected with different combinations of either PTEN or β-gal with PTEN, myr-Akt or β-gal adenoviruses to maintain equal viral load. Over-activation of Akt dramatically reduced the increase in PlGF release stimulated by PTEN-mediated blockade of the PI3K pathway, demonstrating that Akt is the key downstream effector regulating PlGF expression (Fig. 3C). Moreover, the involvement of Akt activity in the hyperglycaemic induction of PlGF was confirmed by the over-expression of myr-Akt, which inhibited hyperglycaemia-induced PlGF release (Fig. 3D).

Akt-1 is the predominant isozyme of Akt in the vasculature and is involved with the regulation of several IGF-1 target genes and implicated in various pathologies^6^. The siRNA–mediated knock-down of Akt-1 in HUVEC (Fig. 3E) significantly increased PlGF secretion into the culture medium (Fig. 3F). In contrast to normoglycaemia and the osmolarity control, the loss of Akt1 did not result in any further increase of PlGF secretion compared with control siRNA treated cells under hyperglycaemic conditions (Fig. 3G). Collectively, our findings show that Akt is a negative regulator of PlGF production in endothelial cells.

### FOXO1 promotes PlGF expression in endothelial cells following hyperglycaemia

**Figure 4:**
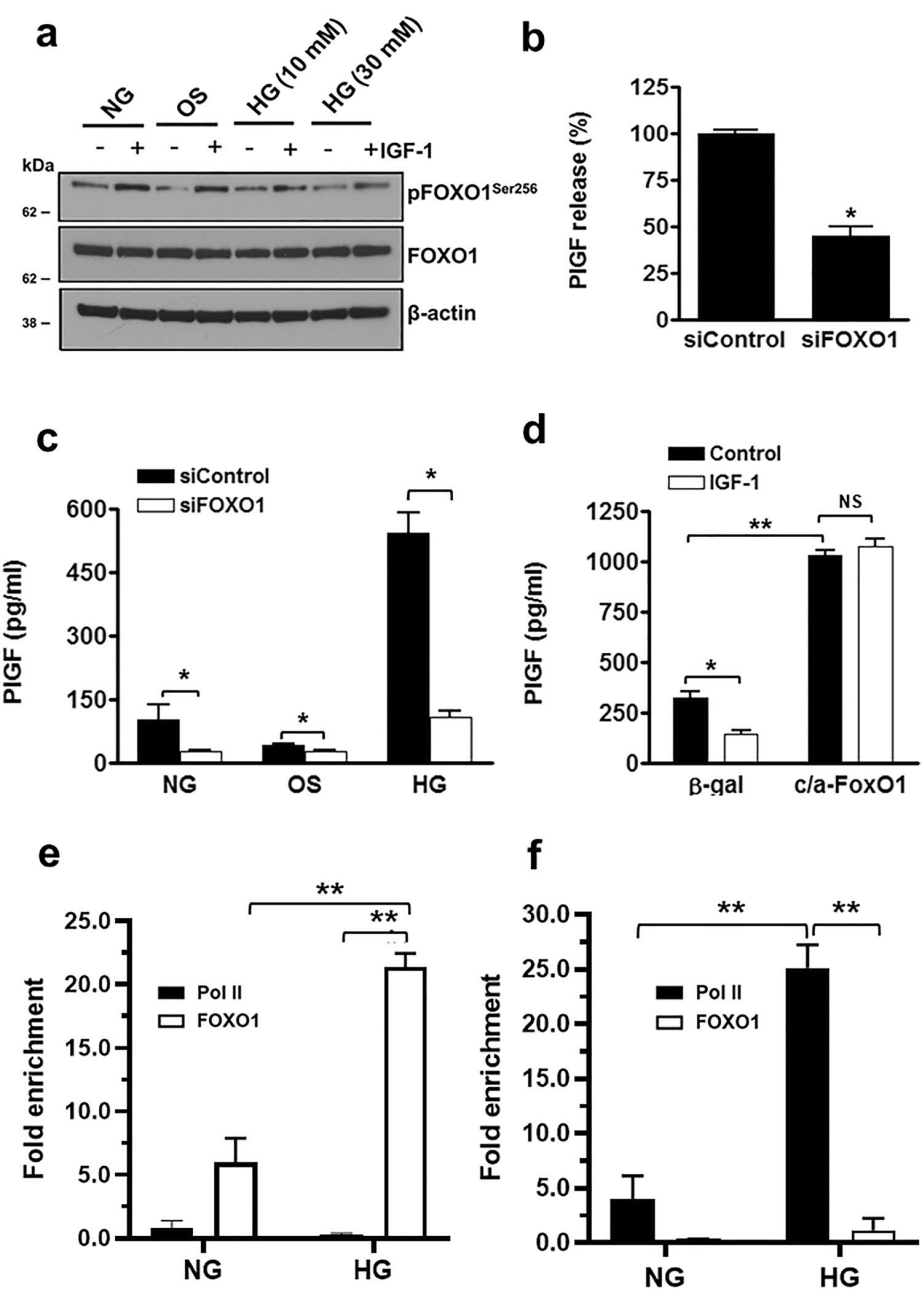
Increased FOXO1 activity induces PlGF expression and release. (**a**) Confluent HUVEC were maintained in normoglycaemia (NG = 5 mM l-glucose), hyperglycaemia (HG = 10, or 30 mM d-glucose) or osmolarity control medium (OS = 5 mM d-glucose + 25 mM l-glucose) and then stimulated with IGF-1 (10 ng/ml) for 10 min, and cell lysates Western blotted for phospho-FOXO1^ser256^, FOXO1 and β-actin (reused from Fig. 1d), with unedited blot images in Supplementary Fig. S5. (**b**) HUVEC were electroporated with FOXO1 (siFOXO1) or control (siControl) siRNAs PlGF secretion into the culture medium quantified by ELISA. (**c**) HUVEC were cultured in NG, HG, or OS for 24 h and stimulated with IGF-1 (10 ng/ml) for 10 min. (**d**) HUVEC infected with ca-FoxO1 and Ad-CMV (control) adenoviruses overnight, then incubated with IGF-1 (10 ng/ml) and PlGF quantified in cell supernatants by ELISA. Quantitative PCR detection of FOXO1 and Pol II enrichment to (**e**) the identified FOXO1 DNA binding site in the PlGF promoter and (**f**) region containing the TATAA box proximal to the PlGF transcription start site, relative to negative control DNA region (NegC) using ChIP assays. Results are the mean (± SEM) of ≥ 3 independent experiments and analysed using (**b**–**d**) Student’s t-test and (**e,f**) one-way ANOVA, Tukey’s post hoc test; *P < 0.05, **P < 0.01.

The forkhead box O (FOXO) transcription factors are inversely regulated by Akt activation through phosphorylation, which leads to inhibition of DNA binding and their exclusion from the nucleus^28^. FOXO1 is the predominant FOXO expressed in the vasculature, adipose tissue, liver and pancreas and is closely linked to glucose metabolism and the regulation of insulin/IGF-1-sensitive genes^28^. Consistent with our findings of reduced Akt activity (Fig. 1D), the level of FOXO1 Serine 256 phosphorylation (pFOXO1^Ser256^) decreased in these cells following exposure to hyperglycaemia indicating an increase of FOXO1 activity (Fig. 4A). Treatment of HUVEC with IGF-1 led to large increase FOXO1 phosphorylation/inhibition in normoglycaemia and the osmolarity control. There was a marked reduction in the level of pFOXO1^Ser256^ in cells incubated with IGF-1 in hyperglycaemia (30 mM glucose) (Fig. 4A). These findings are consistent with the reduced level of Akt phosphorylation under these conditions (Fig. 1D). Similar effects on FOXO1 phosphorylation were also observed following infection of HUVEC with adenovirus expressing myr-Akt (Fig. S3). FOXO1 knock-down in HUVEC resulted in a significant decrease in PlGF mRNA expression and protein release demonstrating the importance of FOXO1 activity in basal PlGF release (Fig. 4B and Fig. S4). In contrast, knock-down of the closely related FOXO3A transcription factor did not affect PlGF expression or release (*see* Fig. S4). To confirm the requirement of FOXO1 in the hyperglycaemic induction of PlGF, HUVEC were incubated in medium containing 30 mM glucose following siRNA-mediated FOXO1 knock-down, which abrogated hyperglycaemia-induced PlGF up-regulation (Fig. 4C). Consistent with these findings, the adenoviral-mediated over-expression of constitutively active FOXO1 (caFoxO1) induced a fourfold increase in PlGF production compared with the β-gal control (Fig. 4D). No reduction in PlGF levels were observed following IGF-1 stimulation of caFOXO1 transduced HUVEC in contrast to the significantly reduced PlGF release in the β-gal controls (Fig. 4D).

In silico analysis of the human PlGF gene sequence identified a FOXO concensus DNA binding sequence (containing the core 5’-AAACAA-3’ motif) between − 3414 bp and − 3423 bp upstream of the PlGF transcriptional start site. Enrichment of FOXO1 binding to this site was investigated using ChIP assays performed with qPCR amplification a 118 bp region flanking the putative FOXO1 binding site relative to a negative control DNA region (NegC). As an additional control, FOXO1 enrichment was also examined at a region flanking the TATAA box proximal to the PlGF transcriptional start site, and compared with the degree of DNA polymerase II (Pol II) enrichment at both sites. Endogenous FOXO1 binding was found to be enriched at the FOXO1 site in the PlGF promoter, which increased significantly (~ fourfold) in HUVEC cultured under hyperglycaemic conditions (Fig. 4E). As expected there was no endrichment of Pol II binding at this site irrespective of glycaemic conditions (Fig. 4E). Conversely, as expected significant enrichment of Pol II was detected proximal to the PlGF transcriptional start site under normoglycaemic conditions which increased (sixfold) in hyperglycaemia, consistent with the up-regulation of PlGF expression in hyperglycaemia (Fig. 4F). As expected there was no enrichment of FOXO1 at the transcriptional start site (Fig. 4F), or Pol II at the FOXO1 binding site (Fig. 4E) further demonstrating specificity of the chromatin precipitation. Collectively, these data demonstrate that FOXO1 activity is required for the direct induction of PlGF expression in hyperglycaemia.

### FOXO1 promotes PlGF expression in vivo

**Figure 5:**
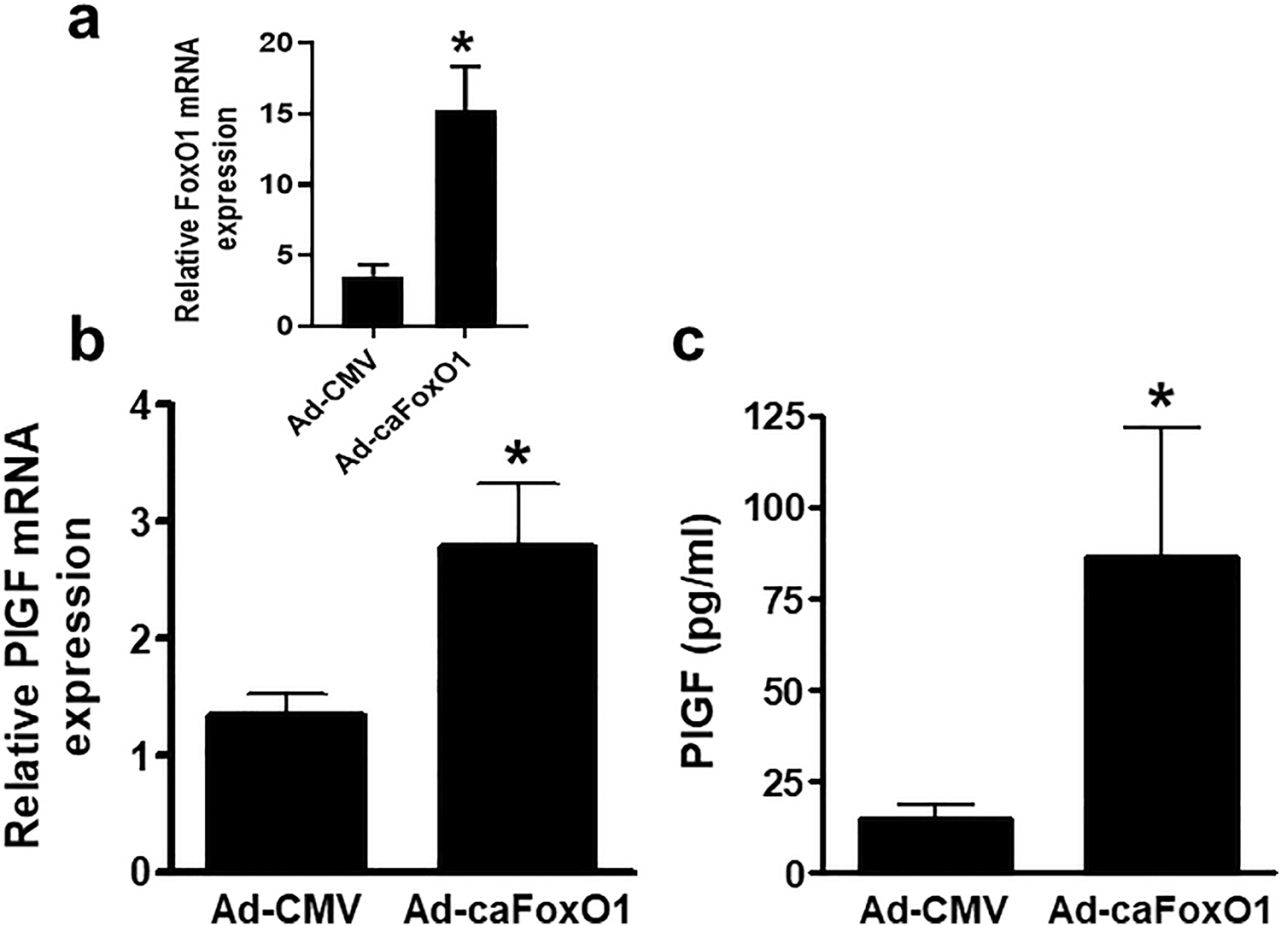
FOXO1 promotes PlGF expression in vivo. FVB/N mice were injected via the tail vein with 2 × 10^10^ infective units of Ad-caFoxO1, or Ad-CMV control virus (8 mice per group). After 48 h the mice were euthanised and blood and liver tissue collected. (**a**) PlGF and FoxO1 mRNA expression quantified in the liver by qPCR and (**b**) circulating PlGF was measured in plasma by ELISA. Results are the mean ± SEM of 8 mice per group; Student’s t test, *P < 0.05.

To determine whether increased FOXO1 activity can regulate PlGF expression in vivo*,* recombinant adenoviruses encoding either caFoxO1 (Ad-caFoxO1) or control empty virus (Ad-CMV) were delivered systemically to FVB/N mice. After 48 h the mice were culled and the levels of FoxO1 and Plgf mRNA in the liver and PlGF protein in plasma, quantified by qPCR and ELISA respectively (Fig. 5). The level of FoxO1 mRNA was found to be increased the livers of the Ad-caFOXO1 group compared with the Ad-CMV control group confirming adenoviral transduction (Fig. 5A). Figure 5B, shows that PlGF mRNA expression was significantly greater in the livers of mice receiving the Ad-caFoxO1 compared with Ad-CMV controls. Furthermore, circulating levels of PlGF in mice treated with caFoxO1 also increased significantly compared with the Ad-CMV control group (Fig. 5C).

## Discussion

The PI3K/Akt pathway is an important regulator of vascular homeostasis and loss of Akt activity results in endothelial dysfunction, which is a characteristic of diabetes, atherosclerosis, and the metabolic syndrome. In this study, we demonstrate that hyperglycaemia directly up-regulates PlGF by suppression of PI3K/Akt signalling resulting in an increase in FOXO1 activity and binding to the PlGF promoter in endothelial cells, and increased FOXO1 activity promotes PlGF expression in vivo. Akt phosphorylation is reported to be reduced by 50% in the internal mammary arteries of type-2 diabetic patients^27^. Similarly, a loss of Akt activity is also reported in models of diabetes^26,29^ and suppression of the PI3K antagonist PTEN restores Akt activity improving insulin sensitivity in mouse models of diabetes^10^. Hyperglycaemia was reported previously to inhibit the PI3K/Akt pathway in vitro in rat retinal endothelial cells^26^. This is consistent with our findings of reduced Akt phosphorylation in HUVEC incubated under hyperglycaemia, both under basal conditions and in the presence of IGF-1, and corresponding changes in FOXO1 phosphorylation/activity in these cells. This resulted in increased FOXO1 binding to the PlGF promoter in HUVEC incubated under hyperglycaemic conditions. These findings are supported by a concomitant increase in Pol II enrichment in the region proximal to the PlGF transcriptional start site. Our data also show that Akt is the major downstream PI3K effector involved in PlGF production. The role of PI3K in PlGF expression was demonstrated by direct manipulation of the PI3K/Akt pathway using pharmacological inhibitors, recombinant IGF-1, or over-expression of wild-type and mutant signalling proteins in endothelial cells. Over-expression of myr-Akt, in the presence of PTEN-mediated inhibition of PI3K, or hyperglycaemia, markedly attenuated PlGF release. Knock-down of Akt1 in endothelial cells produced a similar increase in PlGF production to the general inhibition of Akt activity following over-expression of a dominant-negative Akt mutant, highlighting the importance of the Akt1 isozyme in regulating PlGF production. Akt1 is the predominant form of Akt in the vasculature^30^ and its loss leads to severe atherosclerosis in ApoE^−/−^ mice^6^. It is interesting to note that PCR array data from the same study, indicate that PlGF is up-regulated in mice fed a high fat diet following deletion of Akt1 on an ApoE^−/−^ background^6^.

In contrast to our findings, a study using bovine retinal endothelial cells found that hyperglycaemia induced PlGF expression indirectly via an autocrine VEGF-dependent mechanism, based on the use of a VEGF receptor-2 inhibitor^27^. In our hands, VEGF knock-down in HUVEC did not reduce PlGF expression, as would be expected if PlGF production was secondary to the induction of endogenous VEGF. On the contrary, loss of endogenous VEGF expression was found to promote PlGF expression and secretion significantly (Fig. S2). Furthermore, the observed time-frame of PlGF mRNA induction in response to hyperglycaemia in this study was not consistent with PlGF release dependent on the up-regulation of VEGF expression. However, our findings are in line with more recent studies which show that endogenous VEGF suppresses FOXO1 activity in endothelial cells in an autocrine manner^31,32^. Finally, PlGF up-regulation following stimulation of endothelial cells with recombinant VEGF was reported to be via PKC signalling and independent of PlGF gene transcription^33^.

We have previously examined the level of PlGF production in many human primary and transformed cell types, and apart from trophoblast-derived cell lines (HTR-8/pSVneo, JAR) and thyroid cells the most significant source appears to be both microvascular and large vessel endothelial cells. Indeed, PlGF was reported to be the most abundant VEGF family member expressed in human dermal microvascular endothelium, detected at 100-fold greater levels than the least abundant, VEGF-A^34^. In murine fibroblasts and a human choriocarcinoma cell line, PlGF was reported to be induced by hypoxia through the metal-responsive transcription factor, MTF-1^35,36^. PlGF is switched on selectively in the embryonic kidney by the epithelium-specific BF-2 (FOXD2) transcription factor^37^, and the forkhead-related transcription factor, glial cell missing-1 (GCM-1), regulates PlGF expression in trophoblast cell lines^38,39^. In addition, heme-bound iron has been shown to up-regulate PlGF in erythroid cells via the erythroid krüppel-like factor (EKLF/KLF-1)^40^. However, there is a paucity of information on the control of PlGF expression in endothelial cells. Using siRNA-mediated knock-down or over-expression of constitutively active FOXO1, we demonstrate that PlGF expression is regulated directly by FOXO1 activity in endothelial cells. Furthermore, the increase in PlGF release in response to hyperglycaemia was lost in HUVEC following knock-down of FOXO1, and no IGF-1-mediated suppression of PlGF production was observed in the presence of constitutively active FOXO1. Moreover, FOXO-1 binding was detected at a FOXO-1 consensus DNA binding sequence identified in the PlGF promoter, and enriched ~ fourfold following exposure of the cells to hyperglycaemia. In contrast to our findings, the acute release of PlGF, assessed using protein arrays, from HAEC within one hour was reported to be associated with a loss of FOXO1 activity through C5b-9 complement complex activation of Akt^41^. Conversely, in the same study FOXO1 knock-down actually prevented acute PlGF protein secretion, suggesting a differential regulatory role of FOXO1 in the transcription and secretion/release of PlGF^41^. FOXO1 is the predominant FOXO family member expressed in the vasculature, liver and pancreas and is associated with glucose metabolism and the regulation of insulin sensitive genes^5,42,43^. FOXO1 haploinsufficiency in mice protects against obesity-related insulin resistance^44^ and diet-induced diabetes^45^, and in rat models of diabetes, FOXO1 knock-down in the vitreous reduces retinal endothelial apoptosis in hyperglycaemia^26^. Furthermore, up-regulation of FOXO1 activity in endothelial cells exposed to hyperglycaemia has been identified as a key response promoting oxidative stress through the up-regulation of inducible NO synthase^46^. In addition to decreasing the level of FOXO1 phosphorylation this study showed that hyperglycaemia also reduces the level of FOXO1 acetylation which is associated increased transcriptional activity^46^. The significance of endothelial FOXO1 activity in the promotion of atherosclerosis associated with insulin resistance has also been highlighted^5,47^.

FOXO transcription factors are important modulators of vessel formation, maturation, maintenance and homeostasis^5,42,43,47–49^. Indeed, FOXO1-deficient mice die in utero from embryonic day 9.5, exhibiting vascular defects in the aortic and branchial arches and failure to form blood vessels in the yolk sac^48,49^. FOXO1 was reported to be essential for in vitro vascular formation from embryonic stem cells^50^, and over-expression or knock-down of either FOXO1 or FOXO3A in HUVEC leads to increased endothelial cell migration and capillary-like tube formation on growth factor-reduced Matrigel^50^. Recently the importance of FOXO1 in regulating endothelial cell quiescence has also been highlighted^43,49^. Several genes involved in angiogenesis, vascular remodelling and maintenance/homeostasis, including angiopoietin-2 (Ang-2) are regulated by FOXO1, but not FOXO3A which is associated with apoptosis^42,51^. Similarly, we find that FOXO3A does not regulate PlGF mRNA expression or protein release. Interestingly, microarray data from these studies and others indicate that FOXO1 also regulates VEGFR-1 expression in endothelial cells^42,51,52^. In pathologies, both PlGF and VEGFR-1 are frequently up-regulated in the endothelium^11^, and therefore the increased FOXO1 activity which is often observed in these conditions may provide a common mechanism by which both receptor and ligand are up-regulated to promote PlGF – VEGFR-1 signalling.

Insulin resistance, hyperglycaemia and diabetes are frequently associated with obesity, and are characteristics of the metabolic syndrome. In a cross-sectional survey conducted in three isolated populations in Southern Italy of 1000 men and women, circulating PlGF levels were significantly higher in subjects with the metabolic syndrome and correlated with the number of metabolic criteria in these patients^20^. In addition, circulating PlGF is associated with obesity in children and correlated with troponin levels^22^. PlGF mRNA is expressed in white adipose tissue^53^, and PlGF^(−/−)^ knock-out mice have a lower body weight and fat content than wild-type mice and a lower fat content when maintained on a high-fat diet^54^. Whether this is due to endothelial or adipocyte PlGF production was not fully established, but it suggests that in addition to increased risk of type II diabetes^21^ and CVD^19,23–25^, up-regulation of PlGF in insulin resistance/hyperglycaemia and the metabolic syndrome may also accelerate the development of obesity.

PlGF is a key inflammatory mediator that activates monocytes, promoting migration and inflammatory cytokine and tissue factor expression^11,55,56^. It is suggested that a major component of PlGF activity in pathologies may be through the induction of inflammatory infiltrates, rather than direct activation of the endothelium^11^. However, both PlGF and VEGFR-1 are frequently up-regulated in the endothelium in pathology^11^, and whilst it is established that the majority of endothelial VEGF responses are mediated via VEGFR-2, evidence from our laboratory and others has demonstrated that VEGFR-1 can act as a direct signalling receptor in endothelial cells^57–59^, and PlGF-mediated VEGFR-1 activation leads to distinct endothelial gene expression to that of VEGF^12^. The discovery that the PI3K/Akt/FOXO1 signalling axis regulates PlGF expression in endothelium is likely to be important for its pathological activity in diabetic retinopathy, atherosclerosis, and have wider implications in obesity and the metabolic syndrome, which are all characterised by endothelial dysfunction. Thus, it may represent a unifying pathway by which PlGF signalling may be up-regulated in these conditions and provide the basis for new approaches to limit its activity.

## Methods

### Cell culture

Human umbilical vein endothelial cells (HUVEC) were either isolated, characterised and cultured essentially as described previously^57^ from umbilical cords obtained following informed consent under ethical approval [15-014-014] of the Human Biomaterials Resource Centre, University of Birmingham, UK. Pooled HUVEC from multiple donors were also purchased from PromoCell and human aortic endothelial cells (HAEC) from Lonza and maintained in their endothelial cell growth media and according to the supplier’s recommendations. Endothelial cells were routinely plated on 0.1% gelatin-coated tissue culture flasks/plates and experiments were performed using cells at the third or fourth passage. For experiments to examine PlGF secretion endothelial cells (1 × 10^5^ cells/well) were plated in 24-well plates with 0.5 ml of growth medium to produced confluent monolayers and incubated for 24 h. The medium was then replaced with 0.5 ml MCDB131 (Invitrogen) containing 10% FCS and cells incubated for a further 24 h. The medium was then harvested and centrifuged at 1000×*g* for 5 min and supernatant frozen at − 80 °C prior to ELISA analysis. To examine the effect of hyperglycaemia cells were incubated in MCDB131 medium (Gibco) containing 10% FCS and a final concentration of 5 mM d-glucose (NG = normal glycaemia), 30 mM d-glucose (HG = hyperglycaemia), or 25 mM l-glucose + 5 mM d-glucose (OS = osmolarity control). Recombinant IGF-1 (Peprotech EC, (London, UK) and the PI3 kinase (PI3K) inhibitor, LY294002 (Calbiochem, Nottingham, UK) were added as indicated. MTT assays were performed to ensure that there was no effect of the treatments on cell viability.

### Adenoviruses

The recombinant replication-deficient adenoviruses encoding constitutively active ADA mutant of FoxO1 (Ad-caFoxO1) was a kind gift of Professor D Acilli, Columbia University, NY, USA and dominant-negative Akt (Ad-dn-Akt), myristoylated Akt (Ad-myr-Akt)^60^, PTEN, and PTENc/s were a kind gift of Dr Christopher Kontos, Duke University, USA. All adenoviruses were amplified in HEK-293 cells and isolated from the cells following 4 snap freeze/thaw cycles by CsCl gradient centrifugation and dialysed into 10 mM Tris HCl (pH 8.0), 2 mM MgCl_2_, 4% sucrose and stored at − 80 °C. Purified adenoviruses were titred using the Adeno-X™ Rapid Titer Kit (Takara) and the optimal multiplicity of infection determined by Western blotting as we described previously^57,61^. HUVEC were infected with 50–100 infective units (IFU)/cell and incubated for 18 h in MCDB131 medium containing 5% FCS prior to the experiment.

### Experimental animals

All procedures involving animals were performed in accordance with protocols and regulations approved by the University of Birmingham Animal Welfare Ethics Review Body, under a licence issued in accordance with the Animals (Scientific Procedures) Act 1986, adopting the principles of the 3Rs (Replacement, Reduction and Refinement), and carried out in compliance with the ARRIVE guidelines for animal study (http://www.nc3rs.org.uk/page.asp?id=1357).

FVB/N mice at 10-to-12 weeks of age were housed in a temperature, humidity, and light-controlled room with a 12-h light/dark cycle and allowed free access to water and food. The mice were divided into two age- and sex-matched groups (8 mice per group), housed in different cages, and injected via the tail vein with recombinant adenovirus (2 × 10^10^ IFU) encoding caFoxO1 or Ad-CMV empty control virus. After 48 h the mice were euthanized and blood was collected by cardiac puncture to obtain plasma, and the livers snap-frozen for the preparation of mRNA, by an individual blinded to the original treatment.

### siRNA-mediated gene knock-down

siRNAs targeted to human FOXO1, FOXO3A, AKT1 and VEGF were introduced into HUVEC using the Amaxa Nucleofector HUVEC II kit according (Amaxa) as we described previously^61^, or Lipofectamine RNAiMax transfection reagent (Thermo Fisher) following the manufacturer’s recommended protocol. Following transfection the cells were incubated for 18 h overnight prior to further treatment. The siRNA sequences were selected based on previously reported efficacy as follows: FOXO1, 5′-AAGAGCGUGCCCUACUUCAAG-3′^42,49^; FOXO3A, 5′-AAGAGCUCUUGGU GGAUCAUC-3′^42,49^; VEGF, 5’-AAGGAGUACCCUGAUGAGAUC-3′^62^; Akt1, 5′-AAGGAGGGUUGGCUGCACAAA-3′^63^; A universal control siRNA from Dharmacon was used as a negative control.

### ELISA

Human and mouse PlGF levels were measured in cell supernatants using DuoSet IC ELISA kits according to the manufacturer’s instructions (R&D Systems). Results were expressed either as the mean concentration of PlGF in pg/ml of conditioned medium, or the “mean % release” calculated as a percentage of each sample relative to the untreated control for each experiment.

### RNA isolation and real-time qPCR

The Total RNA plus kit (Norgen Biotek Corp, Canada) was used to isolate RNA from cell and tissues according to the manufacturer’s instructions. Following stimulation, the medium was aspirated and cell monolayers were washed twice with ice-cold PBS and lysed directly in buffer RL. Mouse tissues were disaggregated in ice-cold buffer RL using a Precellys 24 tissue homogenizer set at 2 cycles a 6500 rpm for 20 s with a 30 s interval at 4 °C. Total RNA was extracted and subjected to DNase-1 digestion and ~ 1 μg was reverse transcribed with oligo-dT_18_ primers using the First Strand Synthesis Kit (Promega) for 1 h at 48 °C. Real-time qPCR was performed using a Rotagene RG-3000 (Qiagen) under the following amplification conditions: 95 °C for 10 min, followed by 45 cycles of 95 °C, 10 s; 58 °C, 10 s; 72 °C, 20 s. cDNA samples were amplified in triplicate using SensiMix SYBR green (Bioline) and primers specific for human and mouse PlGF, FOXO1, FOXO3A, VEGF and β-actin (Table S1). PCR products were analysed by 2% agarose gel electrophoresis, sequenced, and post-run melt curve analysis performed to ensure specificity of reactions. The mean threshold cycle (Ct) for each sample was normalised to β-actin levels and fold changes to the experimental control calculated using the ^ΔΔ^Ct method.

### Western blotting

Cell monolayers were washed twice with ice-cold PBS and lysed directly in ice-cold RIPA buffer (Pierce) containing a phosphatase and complete protease inhibitors (Roche) and a total of 30 μg protein of each sample run on 10% SDS-PAGE gels, and blotted onto nitrocellulose or PVDF membranes as described^57^. Membranes were blocked in TBS-T (25 mM Tris–HCl, pH 7.5, 150 mM NaCl, and 0.1% Tween 20) containing 5% (w/v) fat-free milk for 1–2 h and incubated with primary antibodies at the concentration indicated (Table S2) in TBS-T containing 5% BSA overnight at 4 °C. After washing in TBS-T (4 × 10 min), the blots were incubated for 1 h with a 1:10,000 dilution of the appropriate horseradish peroxidase-conjugated antibody (Cell Signalling Technologies) in TBS-T containing 5% (w/v) fat-free milk for 1 h at room temperature and proteins detected by enhanced chemiluminescence. Following the detection of phosphorylated Akt, or FOXO1 membranes were stripped in NaOH (200 mM) for 20 min at room temperature and re-probed for total Akt or FOXO1 protein respectively and β-actin, or α/β tubulin used as a loading control.

### ChIP assays

Endothelial cells were grown to ~ 80% confluence 15 cm culture dishes and treated under the conditions indicated under Cell Culture above. The cells (~ 1 × 10^7^/sample) were fixed/chromatin cross-linked for 10 min using methanol-free formaldehyde (Pierce) added directly to the culture medium to a final concentration of 1%^64^. The formaldehyde was then quenched by adding 2 M glycine to a final concentration of 125 mM and cells washed twice with ice-cold PBS^64,65^. The cells were then scrapped into ice-cold PBS, pelleted and lysed in 50 mM HEPES (pH 8.0) containing, 150 mM NaCl, 10 mM EDTA, 10% glycerol, 0.25% Triton X-100 and complete protease inhibitor cocktail (Roche) and incubated for 10 min at 4 °C with end-over-end rotation^64,65^. Nuclei were collected by centrifugation and resuspended in ice-cold wash buffer (10 mM HEPES, 10 mM EDTA, 0.5 mM EGTA, 0.25% Triton X-100, (pH 8), complete protease inhibitors on a rotary mixer. The nuclei were sonicated on ice using Bioruptor (Diagenode, USA) set on “high” (240 W) with 20–25 cycles of 30 secs ON; 30 secs OFF to give fragmented DNA of ~ 100–300 bp. The sonicated material was then centrifuged for 10 min at 16,000×*g* at 4 °C and the supernatants harvested. Chromatin was diluted with ChIP buffer (25 mM Tris–HCl (pH 8.0), 150 mM NaCl, 2 mM EDTA, 1% Triton X-100, 0.25% SDS, 0.1 mM PMSF and complete protease inhibitors^65^) and 50 μL was kept as the “input” and the rest immunoprecipitated using 10 µg of rabbit anti-FOXO1 (H-128, Santa Cruz Biotechnology, USA), rabbit anti-RNA Pol II (ab26721, Abcam, UK), or isotype-matched IgG as positive and negative controls respectively. The antibody was added with BSA (0.5% final concentration) to 10 µl of protein A/G magnetic beads (Pierce), following washing and resuspension in 100 mM phosphate buffer, and incubated for 2 h at 4 °C on a rotary mixer. The chromatin cross-links were reversed by adding 1 μl of RNAse A (10 mg/ml) with 30 min incubation. Chromatin was subjected to qPCR in triplicate with primers flanking the putative FOXO1 DNA binding sequence identified in the *PlGF* promoter (5’-AGAGCACCAAGCACACCTGTTT-3 and 5’-CAGTGCCCACAACTCAACAC-3’) and proximal to the PlGF transcriptional start site (5’-CCATTCGACATATGCAGGCA-3’ and 5’-CATGATCTAACCGCCTCTGC-3’). A negative control region (NegC; ActiveMotif, UK) was used to calculate the relative enrichment. Input chromatin was used to generate an amplification curve for each primer set to determine the amount of DNA immunoprecipitated with anti-FOXO1, Pol II and isotype-matched IgG control antibodies. Binding data are presented as the mean fold-enrichment at the putative FOXO1 concensus site, or adjacent to the PlGF transcriptional start site, relative to the negative control region (NegC; ActiveMotif) based on the percentage of input obtained.

### Statistical analysis

All data are expressed as the mean ± SD or SEM as indicated. Statistical analysis was performed with GraphPad PRISM using either an unpaired, two-tailed Student’s t-test, or non-parametric one-way ANOVA with Tukey’s multiple comparison post hoc test. A *P* value of < 0.05 was considered statistically significant (*P < 0.05; **P < 0.01).

## Supplementary Information

Supplementary Information.
