# Supplementary material for: Hyperglycaemia up-regulates placental growth factor (PlGF) expression and secretion in endothelial cells via suppression of PI3 kinase-Akt signalling and activation of FOXO1
Source: Sci Rep. 2021 Aug 11;11:16344. doi: 10.1038/s41598-021-95511-8 (PMC8357836; doi:10.1038/s41598-021-95511-8)

SUPPLEMENTARY INFORMATION

Hyperglycaemia up-regulates placental growth factor (PlGF) expression and secretion in endothelial cells via suppression of the PI3 kinase-Akt signaling and activation of FOXO1

Samir Sissaoui, Stuart Egginton, Ling Ting, Asif Ahmed
and Peter W Hewett

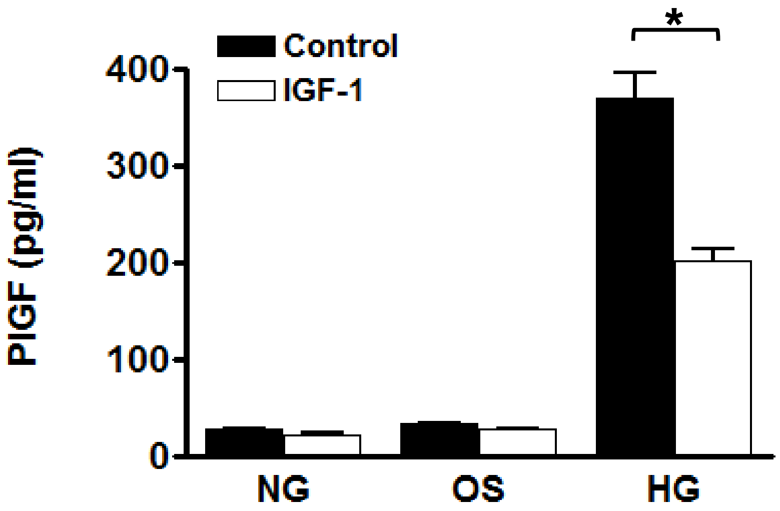

Figure S1: Hyperglycaemia induced PlGF secretion in human aortic endothelial cells (HAEC) is suppressed by IGF-1.

Confluent HAEC were cultured for 24 hours under normolycaemic (NG = 5 mM D-glucose), hyperglycaemic (HG = 30 mM D-glucose) or osmolarity control (OS = 5mM L-glucose + 25 mM D-glucose) conditions in the presence of IGF-1 (10 ng/ml) and the level of PlGF release measured in the medium by ELISA. Results are the mean (± SEM) of 3 independent experiments; Student's t-test *P<0.05.

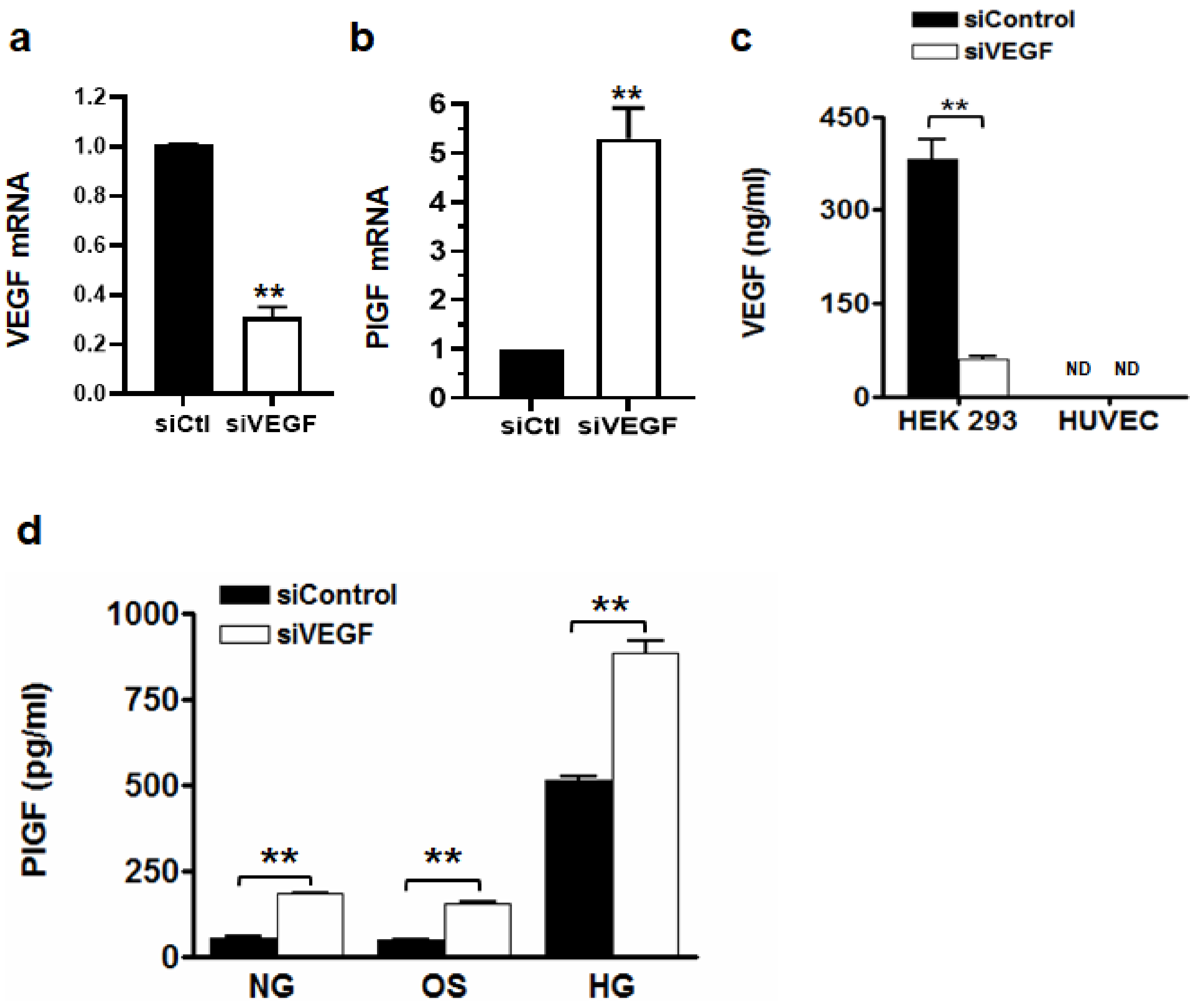

Figure S2: Hyperglycaemia does not up-regulate PlGF expression and secretion via the induction of endogenous VEGF in endothelial cells.

(**a-e**) HUVEC and (**c**) HEK-293 cells were transfected with siRNA targeted to VEGF (siVEGF), or control siRNA (siCtrl/siControl) and incubated overnight. After a further 24 hour incubation in fresh medium, (**a**) VEGF and (**b**) PlGF mRNA expression was quantified by qPCR. (**c**) VEGF levels in HEK-293 cell and HUVEC 24 h supernatants measured by ELISA. *Note*, VEGF was not detectable (ND) in HUVEC cell supernatants using this ELISA. (**d**) PlGF secretion from HUVEC cultured under NG, HG or OS (control) for 24 hours by ELISA. Results are the mean (± SE) of ≥3 independent experiments and analysed by (**a-b**) one-way ANOVA, Tukey's post hoc test, (**c-d**) Student's t-test; *P<0.05, **P<0.01.

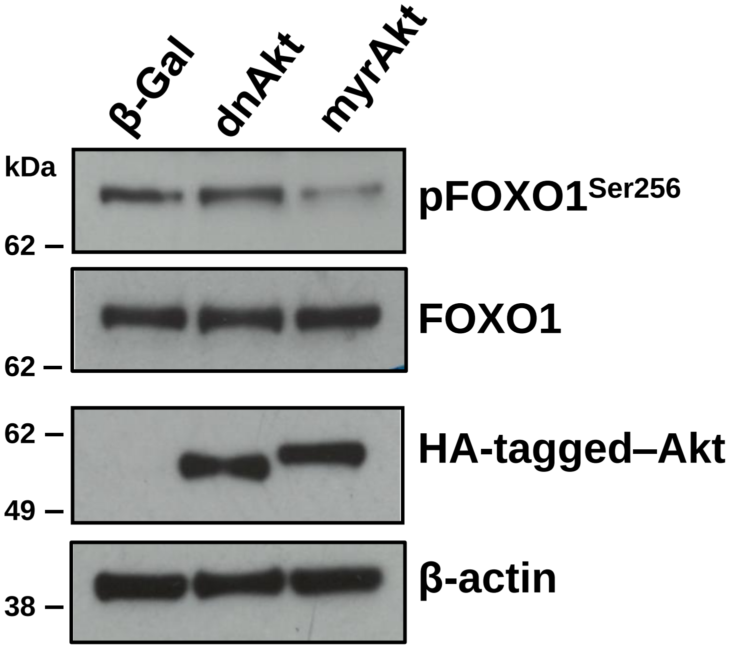

Figure S3: Modulation of FOXO1 activity following infection of endothelial cells with adenoviruses encoding Akt mutants

Endothelial cells were infected overnight with adenoviruses encoding dominant-negative Akt (dn-Akt), constitutively active Akt (myr-Akt) and β-galactosidase (β-gal) in MCDB131 containing 5% FBS). They were then incubated for a further 24 hours in fresh medium (MCDB131 containing 10% FBS) and the cells harvested. Representative Western blots of cell lysates for cytosolic/inactive phosphorylated FOXO1 (pFOXO1^{Ser256}), total FOXO1, detection of the HA-tagged dn-Akt and myr-Akt (HA-tagged-Akt) overexpression in cells and β-actin, as a loading control (*see* Fig. S5 for unedited blot images).

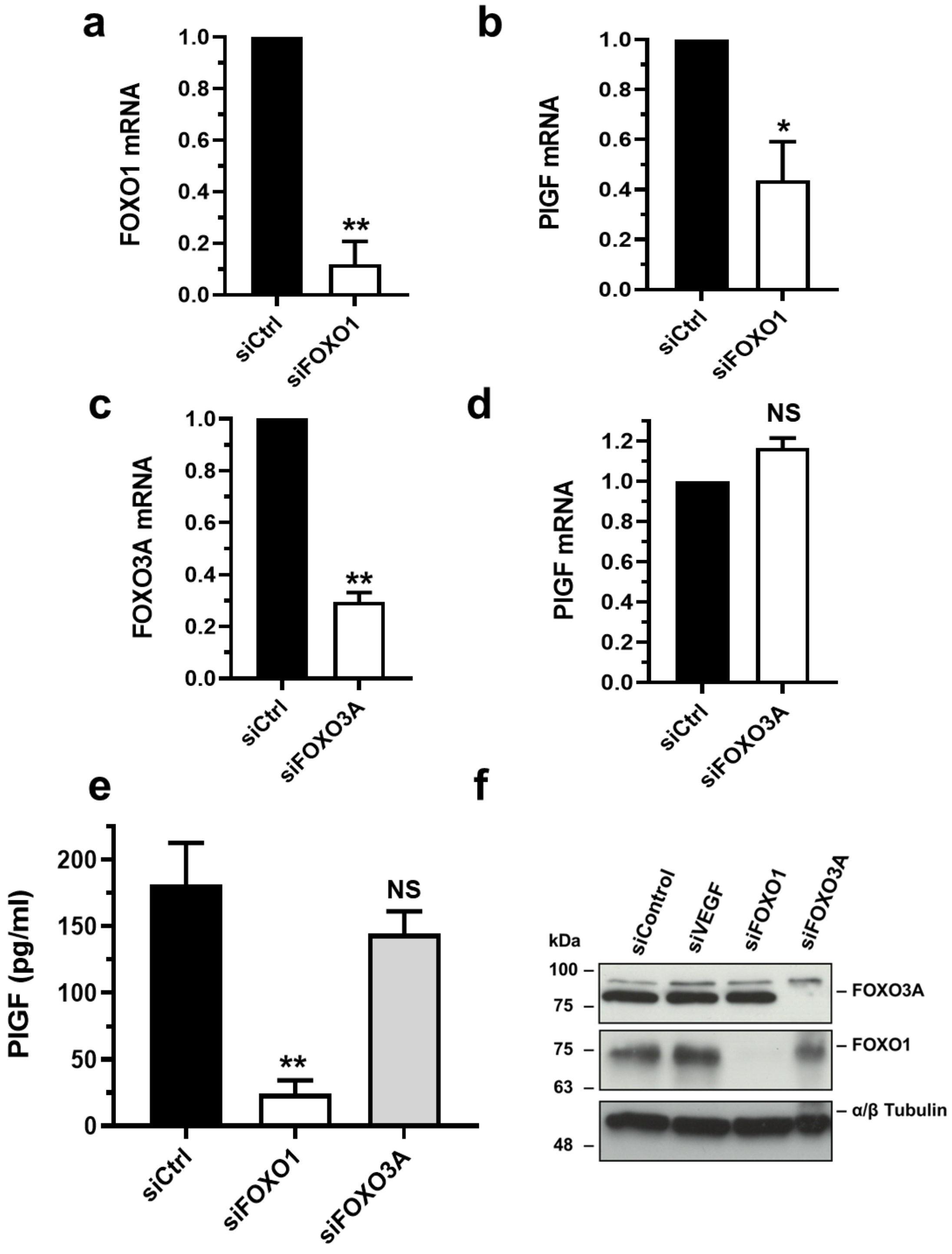

Figure S4: FOXO1 promotes endothelial PlGF expression and release

HUVEC were transfected with FOXO1 (siFOXO1), FOXO3A (siFOXO3A), or control (siCtrl) siRNAs overnight and then incubated in fresh medium (MCDB131 containing 10% FBS) for 24 hours. PlGF secretion quantified and cell lysates Western blotted for FOXO1, and β-actin (*inset*). FOXO1 (**a**), FOXO3 (**c**) and PlGF (**b** & **d**) mRNA expression was quantified by qPCR. (**e**), PlGF was quantified in cell supernatants by ELISA. (**f**) Representative Western blots for FOXO3A and FOXO1 in cells lysates following knock-down with α/β-tubulin as a loading control (*see* Fig. S5 for unedited blot images). l. Results are the mean (± SD) of ≥3 independent experiments and analysed by (**a-d**) one-way ANOVA, Tukey's post hoc test, (**e**) Student's t-test; *P<0.05, **P<0.01.

Target	Primer sequence
PlGF	*sense*: 5’-GCGATGAGAATCTGCACTGT-3’<br>*antisense*: 5’-CTTTAGGAGCTGCATGGTGA-3’
FOXO1	*sense:* 5’-TTGAATTCACCCAGCCCAAACT-3’<br>*antisense:* 5’-GCTACCCCAGGATCAACTGGTG-3’
FOXO3A	*sense:* 5’-TGACTGATATGGCAGGCACCAT-3’<br>*antisense:* 5’-CCGTGCTGTTAAAGGAGCTGGT-3’
VEGF-A	*sense:* 5’-CCTTGCCTTGCTGCTCTACC-3’<br>*antisense:* 5’-ACACTCCAGGCCCTCGTCATTG-3’
β-actin	*sense*: 5’-TCACCCACACTGTGCCCATCTACGA-3’<br>*anti-sense*: 5’-CAGCGGAACCGCTCATTGCCAATGG-3’
mPlgf	*sense*: 5’- GAAGTGGAAGTGGTGCCTTT-3’<br>*antisense*: 5’-CGACTCAGAAGGACACAGGA-3’
mFoxO1	*sense:* *5’*-TTCAATTCGCCACAATCTGTCC-*3’*<br>*antisense:* *5’*-GGGTGATTTTCCGCTCTTGC-*3’*
mβ-actin	*sense*: 5’-GTATGCCTCGGTCGTACCA-3’<br>*antisense*: 5’-CTTCTGCATCCTGTCAGCAA-3’

Table S1: Sequences of the primers used to amplify human and mouse (denoted by “m” prefix) sequences for real-time qPCR analysis.

Antibody to	Species / type	Clone / Cat #	Dilution	Supplier
Akt	rabbit polyclonal	#9461,	1:1000	Cell Signaling Technology
pAkt^{ser473}	rabbit polyclonal	#9271	1:500	Cell Signaling Technology
PTEN	mouse monoclonal	A2B1	1:500	Santa Cruz Bioechnology
FOXO1	rabbit monoclonal	C29H4	1:1000	Cell Signaling Technology
pFOXO1^{ser256}	rabbit polyclonal	#9461	1:1000	Cell Signaling Technology
FOXO3A	rabbit monoclonal	D19A7	1:1000	Cell Signaling Technology
α/β-tubulin	rabbit polyclonal	#2148	1:1000	Cell Signaling Technology
β-actin	rabbit polyclonal	#4967	1:2000	Cell Signaling Technology
Anti-HA	mouse monoclonal	12CA5	1:1000	Roche

Table S2: Antibodies and dilutions used for Western blotting.

Figure S5: Original unedited images of Western blots used to compile the Figures.

S5A: The dashed boxes correspond to the cropped blot images presented in **Figures 1d & 4a.**

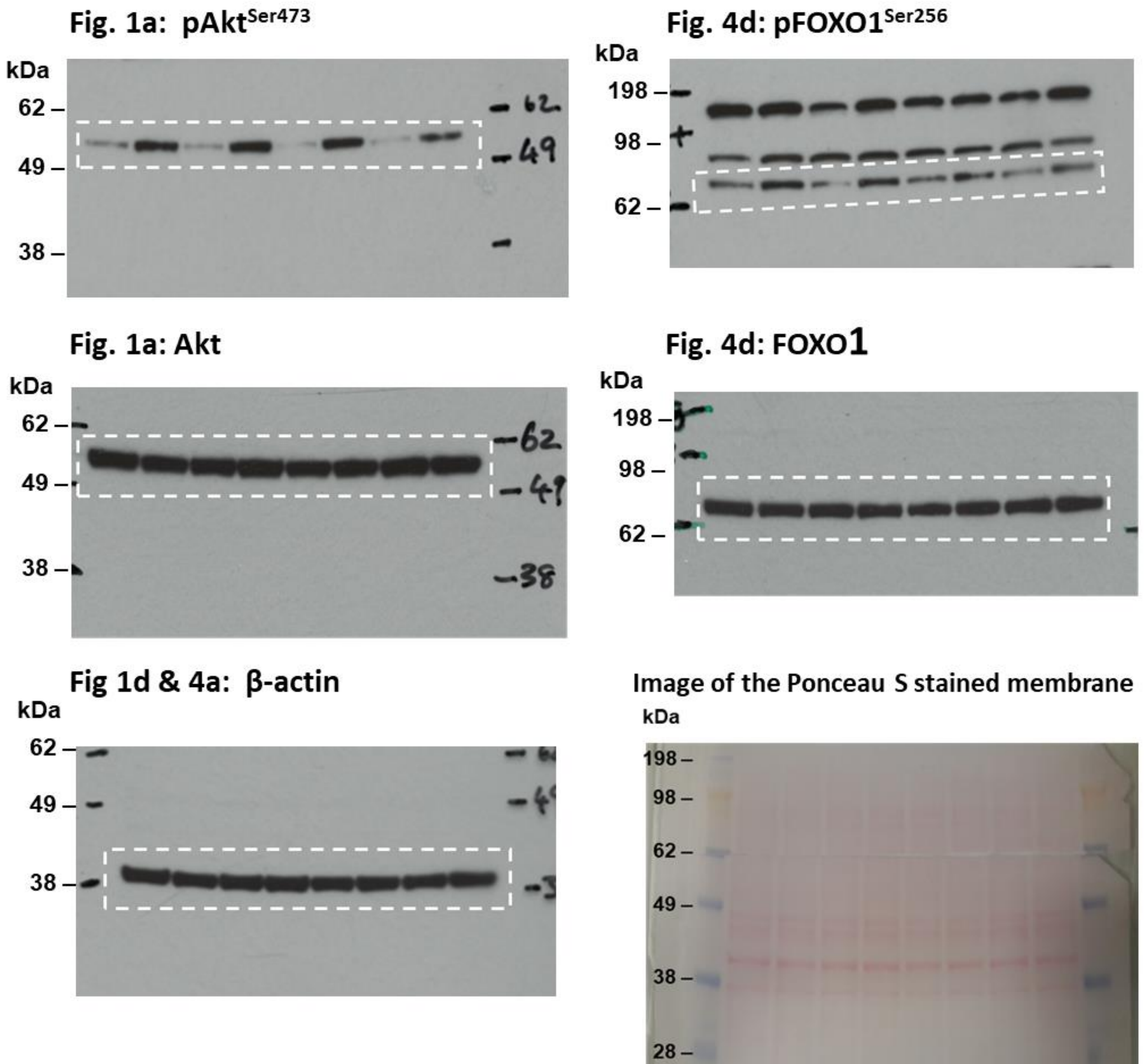

The position of the SeeBlue Plus2 pre-stained MW markers (ThermoFisher) run are indicated.

S5B: Original unedited images of Western blots used to compile **Figures 2d, 2f** & **3f.** The dashed boxes correspond to the cropped blot images presented. The position of the SeeBlue Plus2 pre-stained MW markers (ThermoFisher) run are indicated.

Figure 2d:

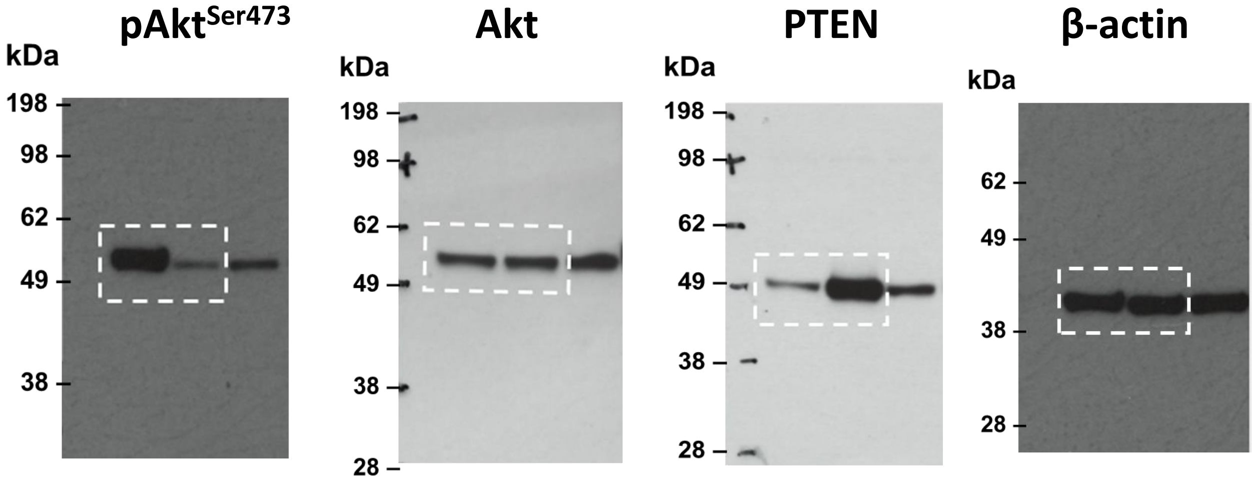

Figure 2f:

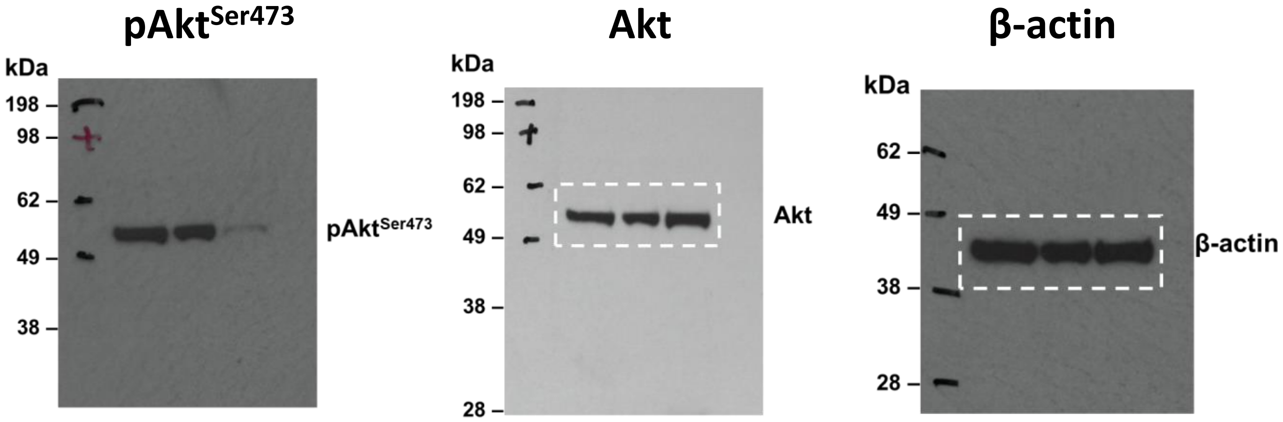

Figure 3f:

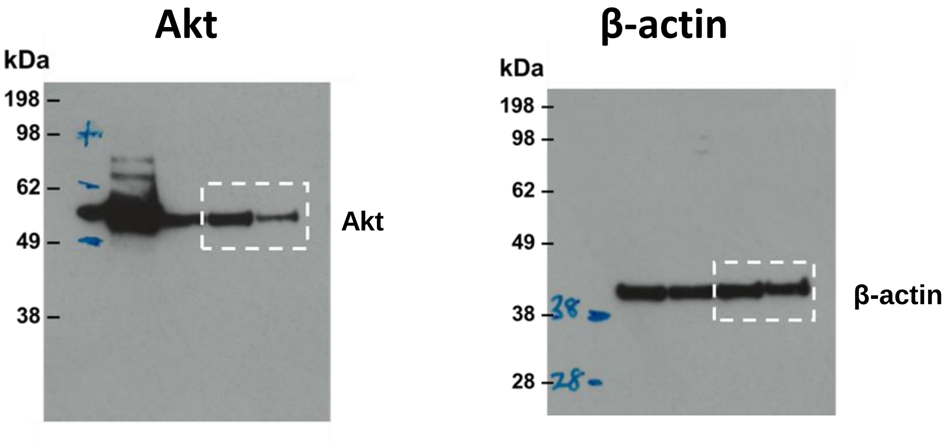

S5C: Original unedited images of Western blots used to compile **Supplementary Figures S3** & **S4f**. The dashed boxes correspond to the cropped blot images presented. The position of the SeeBlue Plus2 (ThermoFisher) (**Fig. S3**) or BLUeye (Cleaver Scientific) (**Fig. S4f**) pre-stained MW markers run are indicated.

Figure S3:

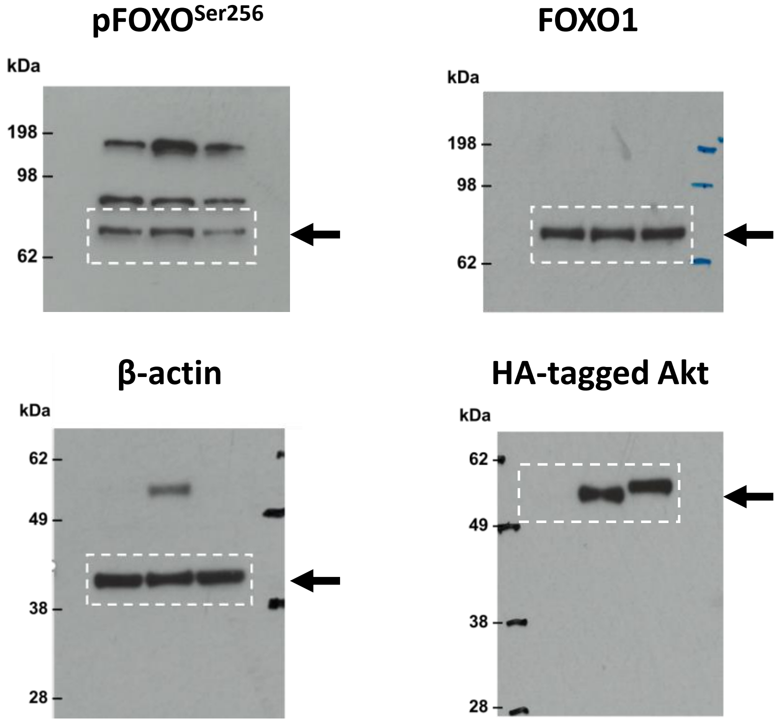

Figure S4f:

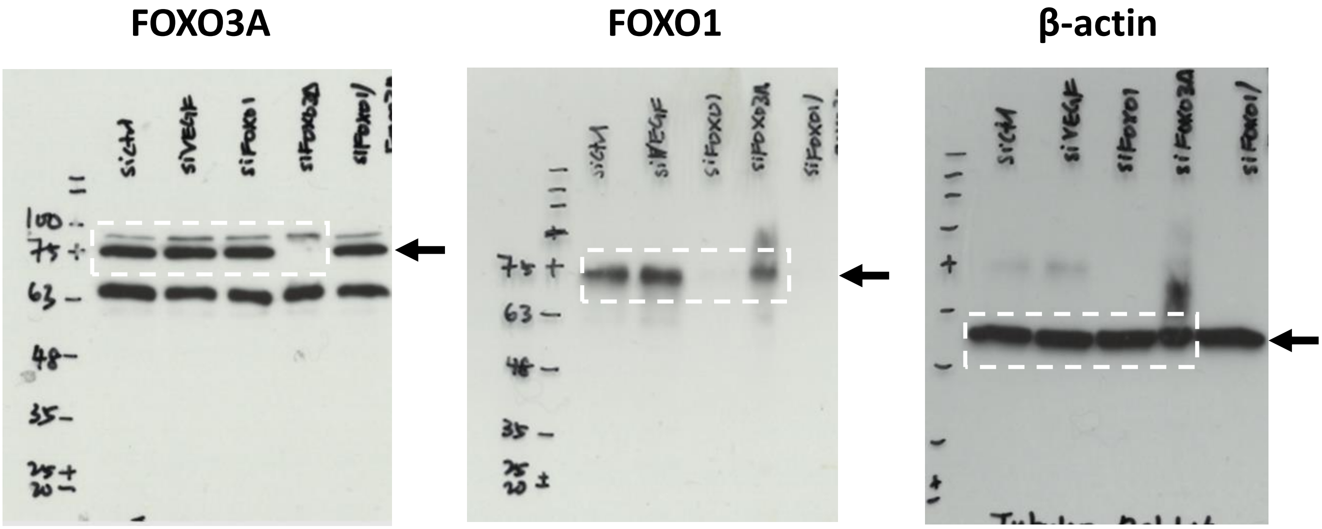

Supplement: Supplementary file 1 — Supplementary Information. [file 41598_2021_95511_MOESM1_ESM.pdf]
